# Entomological surveillance following a long-lasting insecticidal net universal coverage campaign in Midwestern Uganda

**DOI:** 10.1186/s13071-015-1060-6

**Published:** 2015-09-17

**Authors:** MEH Helinski, A. Nuwa, N. Protopopoff, M. Feldman, P. Ojuka, DW Oguttu, TA Abeku, S. Meek

**Affiliations:** Malaria Consortium, Plot 25, Upper Naguru East Rd, PO Box 8045, Kampala, Uganda; Malaria Consortium, Development House, 56-64 Leonard Street, London, EC2A 4LT UK; Vector Control Division, Ministry of Health, P.O. Box 1661, Kampala, Uganda; Present affiliation: Department of Disease Control, London School of Hygiene & Tropical Medicine, Keppel Street, London, WC1E 7HT UK; Present affiliation: Independent public health consultant, #303, 30 River Walk Gardens, London, SE10 0GA UK

**Keywords:** Uganda, Malaria, *Anopheles*, EIR, LLIN, *Kdr*, Universal coverage campaign

## Abstract

**Background:**

A universal coverage campaign (UCC) with long-lasting insecticidal nets (LLINs) was implemented in four districts in Midwestern Uganda in 2009–2010. Entomological surveys were carried out to monitor changes in vector density, behaviour and malaria transmission following this intervention.

**Methods:**

*Anopheles* mosquitoes were collected using CDC light traps quarterly and human landing catch twice a year in four sites. Collections were done at baseline before the campaign and over a three-year period following the campaign. *Plasmodium falciparum* circumsporozoite enzyme-linked immunosorbent assays were performed. A subset of anophelines were molecularly identified to species, and *kdr* L1014S frequencies were determined.

**Results:**

The prevailing malaria vector in three sites was *Anopheles gambiae s.l.* (>97 %), with *An. funestus s.l.* being present in low numbers only. *An. gambiae s.s.* dominated (> 95 %) over *An. arabiensis* within *A. gambiae s.l.* In the remaining site, all three vector species were observed, although their relative densities varied among seasons and years. Vector densities were low in the year following the UCC but increased over time. Vector infectivity was 3.2 % at baseline and 1.8 % three years post-distribution (*p* = 0.001). The daily entomological inoculation rate (EIR) in 2012 varied between 0.0-0.98 for the different sites compared to a baseline EIR that was between 0.0-5.8 in 2009. There was no indication of a change in indoor feeding times, and both *An. gambiae s.l.* and *An. funestus s.l.* continued to feed primarily after midnight with vectors being active until the early morning. *Kdr* L1014S frequencies were already high at baseline (53–85 %) but increased significantly in all sites over time.

**Conclusions:**

The entomological surveys indicate that there was a reduction in transmission intensity coinciding with an increase in use of LLINs and other antimalarial interventions in areas of high malaria transmission. There was no change in feeding behaviour, and human-vector contact occurred indoors and primarily after midnight constantly throughout the study. Although the study was not designed to evaluate the effectiveness of the intervention compared to areas with no such intervention, the reduction in transmission occurred in an area with previously stable malaria, which seems to indicate a substantial contribution of the increased LLIN coverage.

## Background

Sub-Saharan Africa has seen an impressive scale up in coverage of malaria prevention tools and treatment, which contributed to an overall reduction in malaria morbidity and mortality in recent years [1, 2]. Uganda is among the countries with the highest burden for malaria, and 90 % of the 37.6 M population resides in high endemicity areas [2]. The main vectors in Uganda are *Anopheles gambiae s.s.*, *An. arabiensis*, and *An. funestus s.s*; although relative species composition varies considerably between areas. The country is scaling up a number of interventions aimed to reduce malaria prevalence and improve case management. Universal coverage (i.e., one net for every two people as defined in the context of Uganda) with long-lasting insecticidal nets (LLINs), and indoor residual spraying (IRS) in selected districts, are the main vector control interventions being implemented.

Insecticide treated nets reduce vector contact and mosquito life span, leading to reductions in both vector density and infectivity and thus transmission intensity [3], and ultimately malaria morbidity and mortality [4]. One of the methods to measure malaria transmission intensity and any changes as a result of interventions is through the entomological inoculation rate (EIR), which is the number of infective bites a person receives over a time period. Besides modifying transmission intensity, large-scale LLIN use may impact anopheline biting time and location which could undermine control efforts [5]. A number of studies reported behavioural changes as a result of insecticide treated net (ITN) introduction to early evening and morning feeding when people are not protected by nets [6–8]. In addition, changes to more outdoor feeding were observed in some studies [9, 8].

The extensive use of LLINs, coupled with agricultural use of pesticides, has led to resistance of mosquito vectors to pyrethroid insecticides in sub-Saharan Africa [10], although the impact of resistance on LLIN effectiveness is not fully understood [11]. Phenotypic resistance to pyrethroid insecticides is widespread in *An. gambiae s.s.* from Uganda while for *An. arabiensis* varying levels of resistance are observed [12–14]. The knock-down resistance (*kdr)* L1014S mutation has been detected in *An. gambiae s.s.* from Uganda from 2001 onwards [14], sometimes at high frequencies [13]. In contrast, only low *kdr* L1014S frequencies (<1 %) were detected in *An. arabiensis* [13]. The *kdr* L1014F mutation was rare in *An. gambiae s.s* and absent in *An. arabiensis* collected from Uganda [12, 13]. Besides *kdr*, evidence of metabolic resistance mechanisms were observed in *An. gambiae s.s.* [13, 14]. Additionally, pyrethroid resistance was observed in *An. funestus s.s.*, mediated by metabolic resistance mechanisms [15].

The entomological studies performed here were part of the Pioneer project, which was implemented in five districts in Midwestern Uganda from April 2009 to May 2014. Prevalence of malaria in children under five in the area was between 42.7-50.7 % in the Malaria Indicator Survey of 2009 [16]. The project’s focus was on systemic malaria control working through existing structures, and strived to increase both supply and demand for quality malaria prevention, diagnostics and treatment tools [17]. As part of this project, a LLIN universal coverage campaign (UCC) using Olyset® nets, treated with permethrin [18] was conducted between December 2009 and March 2010 in all districts with the exception of Kibaale, where the campaign was only conducted in three sub-counties in August 2010 due to a shortage of nets. Household ownership of at least one ITN was 79 % approximately 1.5 years following distribution, compared with 22 % prior to the campaign; while the proportion of children under five that slept under an ITN increased from 13.7 to 59.6 % (Malaria Consortium, unpublished data). Besides the LLIN campaign, the project implemented a range of behaviour change communication activities to increase awareness and promote health care seeking behaviour. Furthermore, the area benefitted from increased availability of artemisinin-based combination therapies through the integrated community case management program, and improved malaria diagnostics at facilities through the introduction of rapid diagnostic tests and strengthening of malaria microscopy [19].

The present study aimed to gather data on entomological variables following the implementation of LLINs and other interventions to monitor changes in vector density, biting rates and infection rates, and to determine whether vector behaviour was in line with the intended use of the interventions. In addition, trends of *kdr* resistance genotype levels were studied following the LLIN distribution. The study was not designed to evaluate effectiveness of the interventions compared to absence of such interventions, so the entomological monitoring was restricted to the LLIN campaign target areas.

## Methods

### Study sites

**Fig. 1:**
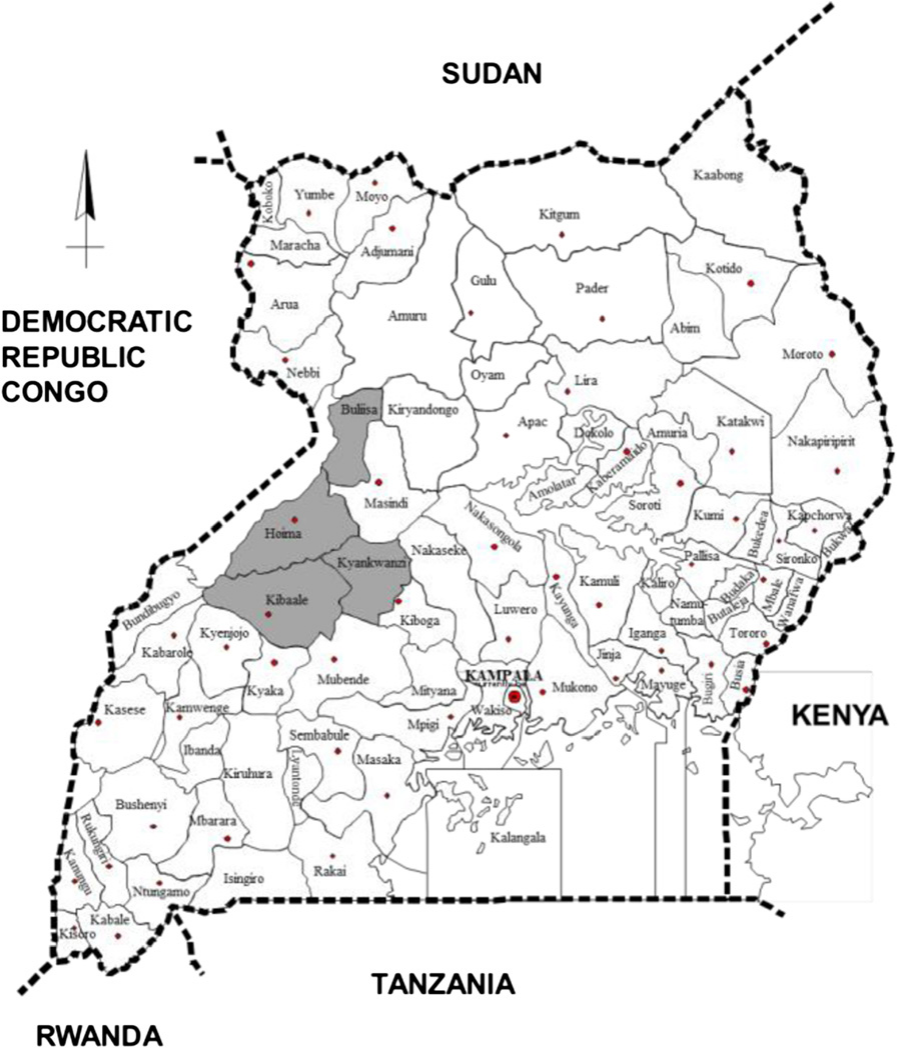
Map of Uganda with study districts highlighted

The entomological surveillance was carried out in one site each in the districts of Buliisa (N1°49' 7.78" E31°19' 29.71"), Hoima (N1° 25' 22.08" E31° 18' 24.12"), Kyankwanzi (N1° 7' 29.28" E31° 35' 53.88") and Kibaale (N1° 3' 54.72" E30° 41' 44.52") located in Midwestern Uganda (Figure 1). The elevation ranged from 621 m in Buliisa to 1281 m in Kibaale. Mosquito collections were done in the same sites for the duration of the study period except in Kibaale where the study site was changed from Rubirizi village to Muhorro (N0° 55' 0.21" E30° 45' 28.48") from the third round (May 2010) onwards as the former site did not benefit from the UCC. All four study sites experience two rainy seasons in the months of March-May, and September-November. In Kibaale district, the August 2012 collection was not performed due to an Ebola outbreak in this district resulting in restrictions on movement.

### Mosquito collections & processing

Mosquito collections started in November 2009 (baseline, round 1) prior to the distribution of nets and continued until February 2013 (round 14). Mosquitoes were collected using two methods: CDC light trap collection (LTC) and human landing collection (HLC) using mouth aspirators. In each of the four sites, collections were made in six households; three houses were used for HLC while the other three were used for LTC. Houses selected represented typical constructions in the area, and the majority of houses had mud walls, sand floors, and iron roofing.

In each study site light traps were deployed quarterly in February, May, August, and November for six consecutive nights. Light traps were installed at the head end of the bed, and occupants were covered by an untreated net. Light traps were installed in bedrooms at 18:00 h and removed at 06:00 h the next morning. HLC were performed every six months (May and November) for six nights. At each house, indoor collections were done from 18:00 to 06:00 h and outdoor collections from 18:00 to midnight. Collectors worked in teams of three per house, and each collector worked a shift of three hours, to a maximum of two shifts per collector per night. Prophylaxis (doxycycline) was made available to collectors assigned to HLC collections.

Culicine females were counted and discarded. All anopheline mosquitoes were separated by sex and females identified morphologically in the field and classified into the *An. gambiae* complex, the *An. funestus* complex, and “other anophelines”. Specimens were packed individually in capsule tubes and kept in plastic bags with silica gel for further processing. Most samples from the collection in November 2010 could not be analysed due to loss of the specimens.

### Ethical approval

Ethical clearance for this activity was obtained from the Uganda National Council for Science and Technology. Written informed consent was obtained from household owners while oral informed consent was obtained from collectors assigned to HLC collections.

### Laboratory testing

#### Sporozoite assays

The majority of specimens from HLC (i.e., 82 %) were analysed to detect *Plasmodium falciparum* circumsporozoite protein using the sandwich enzyme-linked immunosorbent assay (ELISA) following set procedures [20]. The monoclonal antibody (MAb-Pf) and positive control were obtained from the Centers for Disease Control and Prevention (CDC) in Atlanta, USA. For each specimen, only the head and thorax were tested. Results were read visually against the positive control. Analyses were carried out at the Vector Control Division (VCD) laboratory, Ministry of Health, in Kampala.

#### Molecular analyses

Molecular analyses on a subset of samples were performed. Samples were analysed for species identification for the *An. gambiae s.l.* or *An. funestus s.l.* complex based on original field identification. An attempt was made to obtain representative subsamples from the different sites, rounds and collection methods for *An. gambiae* s.l. specimens. To this end, for collections containing less than 500 samples, up to 20 samples were randomly selected; while for collections containing more than 500 samples, on average 5 % of samples were randomly taken. A random subset of *An. gambiae s.s and An. arabiensis* were further analysed for the *kdr* L1014S mutation; samples selected were representative for the different sites and collection years. A limited number of *An. gambiae s.s and An. arabiensis* were also analysed for the *kdr* L1014F mutation. Genomic DNA was extracted from body parts (leg or wings) using the Chelex method [21] and was stored at −20 °C until use. Real time polymerase chain reaction (PCR) TaqMan assays were used to distinguish between the two sibling species *An. gambiae s.s* and *An. arabiensis* [22] and to detect *kdr* mutations [23]. Genotyping results were analysed using MXPro software (Agilent technologies, Stratagene, USA). Some samples could not be reliably classified and were removed from the analyses. A conventional PCR was used to distinguish members of the *An. funestus* complex [24, 25], and samples were randomly selected from different sites and rounds. Analyses were done at PAMVERC Laboratory in Moshi, Tanzania.

### Data analyses

All data were entered and validated in Epidata 3.1 (EpiData Association, Denmark). Data were analysed in STATA version 12 (Statacorps, USA).

LTC data was averaged over the number of collection nights and houses in each site to arrive at a mean anopheline density per house per night per survey. A negative binomial regression was used to compare the number of *An. gambiae s.l.* (all sites) and *An. funestus s.l.* (Buliisa site only due to low numbers collected in other sites) collected by light traps before and after the UCC for each site. Comparisons were made for the same collection month because of seasonality observed. In all sites but Kibaale, comparisons were made for the November months of 2009 and 2010. In Kibaale, the comparison was made for the month of May in 2010 and 2011 as the first two collections were done in a different site and the nets were only distributed in August of 2010. We used a negative binomial regression because it is robust to the presence of a large number of zero counts in light trap collections [26, 27].

Human biting rates (i.e., the number of bites per person per night) were calculated for each site and each survey taking into account the number of collectors working simultaneously, the number of collection nights, and the assumed night time behaviour of the local populations. It was assumed that an average villager in each of the sites spends 1 h on average outdoors between 18:00 h and 22:00 h, and all villagers are indoors after 22:00 h [28]. Sporozoite rates were calculated by dividing the number of positive mosquitoes over the total number tested. EIR figures were calculated by multiplying human biting rates with sporozoite rates.

Wilcoxon signed-rank tests were used to compare the number of anophelines collected by indoor or outdoor collection during the period of 18:00–00:00 h. For Kyankwanzi, Hoima and Kibaale samples, comparisons were made for *An. gambiae s.l.* only due to the low number of *An. funestus s.l.* observed, while in Buliisa comparisons were made separately for each species. *Kdr* L1014S genotype frequencies over survey years for *An. gambiae s.s.* were compared with Pearson chi-square tests by site.

## Results

### Species composition

A total number of 18,437 anopheline females and 35,133 culicine females were collected during the course of the study. The predominant malaria vector overall was *An. gambiae s.l.*, (91 %) while *An. funestus s.l.* constituted 8 % of the collections (Table 1). A small number of other anophelines (1 % of the total collections) were observed but these were not identified further.

**Table 1:** Total number of *An. gambiae s.l.* and *An. funestus s.l.* mosquitoes collected at the four study sites by human landing collection (HLC) and light trap collection (LTC)

Site	*An. gambiae s.l.*				*An. funestus s.l.*			
	HLC		LTC	Total	HLC		LTC	Total
	Indoor	Outdoor			Indoor	Outdoor		
	(18:00–06:00)	(18:00–00:00)	(18:00–06:00)		(18:00–06:00)	(18:00–00:00)	(18:00–06:00)	
Hoima	776	116	649	1,541	28	4	12	44
Kyankwanzi	9,097	705	2,142	11,944	20	5	1	26
Buliisa	945	194	466	1,605	439	32	901	1,372
Kibaale	345	80	1,274	1,699	0	0	3	3
Total	11,163	1,095	4,531	16,789	487	41	917	1,445

Times of collection are indicated

The prevailing malaria vector in each site except Buliisa was *An. gambiae s.l* (> 97 %), with *An. funestus s.l.* being present in low numbers only. In Buliisa, *An. funestus s.l.* made up 46 % of the collections. There was substantial variation in vector numbers among the four sites: 71 % of the *An. gambiae s.l.* mosquitoes were collected from the Kyankwanzi site, whereas similar numbers were observed at the other three sites.

The four collection sites represented different species compositions for the *An. gambiae* complex. In Kyankwanzi, Hoima and Kibaale *An. gambiae s.s.* was almost exclusively found, and only a small number of *An. arabiensis* was observed, mainly from outdoor collections (0-4 %; Table 2). *An. arabiensis* was more common in Buliisa, where between 36-59 % of analysed mosquitoes were *An. arabiensis*.

**Table 2:** Percent *An. gambiae s.s* and *An. arabiensis* found following molecular species identification, by collection method and site

Study site	*N*	Indoor HLC		Outdoor HLC		LTC	
		*An. gambiae s.s.*	*An. arabiensis*	*An. gambiae s.s.*	*An. arabiensis*	*An. gambiae s.s.*	*An. arabiensis*
Hoima	308	99.3	0.7	95.7	4.3	100.0	0.0
Kyankwanzi	915	98.6	1.4	94.2	5.8	96.2	3.8
Buliisa	296	64.5	35.5	40.8	59.2	60.9	39.1
Kibaale	271	100.0	0.0	100.0	0.0	98.9	1.0

N is the number of samples that successfully amplified

The *An. funestus* complex was mainly analysed from Buliisa, as in other sites few specimens were collected. *An. funestus s.s.* was most common and 96 % of samples in Buliisa belonged to this species (*N* = 161). A small number of other sibling species were observed; i.e., *An. leesoni* (4 specimens), *An. rivulorum* (1 specimen), and *An. parensis* (1 specimen). The majority of these were observed in the outdoor HLC collection. In Hoima all samples tested were *An. funestus s.s.* and came from LTC and indoor HLC (*N* = 18), while in Kyankwanzi the only sample positively identified was also *An. funestus s.s.*

### Light trap collections

**Fig. 2:**
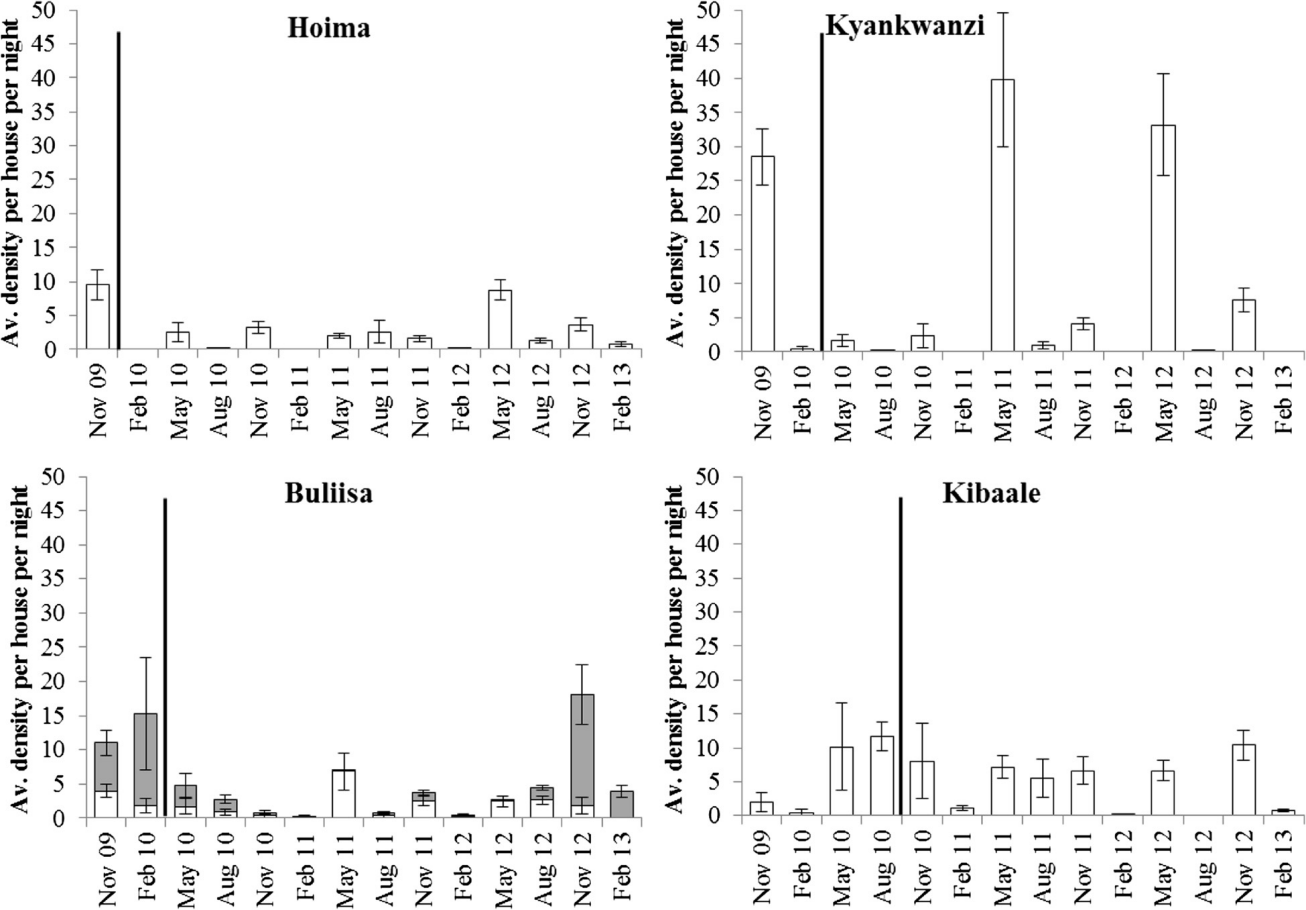
The average number (± SEM) of *An. gambiae s.l.* (white bars) and *An. funestus s.l.* (grey bars; only for Buliisa) per house per night collected by light traps. In Kibaale the first two collections were done in a different site than all subsequent rounds. No collections were done in Kibaale in Aug 12 due to an Ebola outbreak. Lines indicate when nets were introduced in the entire district, with the exception of Kibaale where nets were only distributed in three sub-counties

The average number of *An. gambiae s.l.* collected per house per trapping night was significantly lower compared to baseline densities in the first year after the UCC in Hoima (z = −2.70; *p* = 0.007), Kyankwanzi (z = −4.32; *p* < 0.001) and Buliisa (z = −3.88; *P* < 0.001; Fig. 2) when data for the month of November was compared. *An. funestus s.l.* densities in Buliisa declined the year after the UCC (z = −3.69; *p* < 0.001), yet numbers increased again in 2012. In Kibaale, vector densities remained similar throughout the collection period yet in general few anophelines were trapped. Additionally, the first two surveys were conducted in a site different from the one used in later surveys, which may explain the low densities observed during the initial two rounds.

A marked seasonality was observed in Kyankwanzi with highest numbers of vectors collected in November and May. Vector densities in Hoima were sustained at a low level throughout 2010 and 2011 but vector density increased in 2012. In Kyankwanzi, a similar pattern was observed and densities in May of 2011 and 2012 were higher than during the baseline collection in 2009. *An. gambiae s.s.* was the dominant vector collected by light trap in all sites, except in Buliisa where *An. arabiensis* was more common (i.e., 39 %; Table 2). Vector densities in Buliisa showed an interesting pattern with *An. gambiae s.l.* being more prevalent than *An. funestus s.l.* in 2011, while the opposite trend was observed in 2012 (Fig. 2).

### Human landing collections

The human biting rate (HBR), expressed as the number of bites per person per night, was highest in Kyankwanzi in November of 2009 for *An. gambiae s.l.* with 197.1 bites per person per night (Table 3). The HBR fell sharply in Kyankwanzi to 7.5 and 2.9 bites per person per night in May and November of 2010, respectively. In subsequent years the HBR increased, although values did not reach the baseline figure. A similar trend was observed in Hoima, although the HBR was much lower overall compared to Kyankwanzi. In Kibaale, the HBR decreased in November of 2010 following the UCC in August of 2010, but was back at similar levels in November of 2012. In Buliisa, the HBR for *An. gambiae s.l.* showed an increase in May 2011, similarly to the increase seen in the density collected by light trap (Fig. 2). *An. funestus s.l.* HBRs fell from 10.2 bites in 2009 to 1.2 and 0.1 bites in May and November of 2010, respectively, but in 2012 the HBR was similar to baseline levels (Table 3).

**Table 3:** Human biting rates (HBR; number of bites per person per night) for HLC by site and survey round. Results are presented for *An. gambiae s.l.* for all sites and for *An. funestus s.l.* for the Buliisa site only

Human biting rates	*An. gambiae s.l.*						
Year	2009	2010		2011		2012	
Month	Nov	May	Nov	May	Nov	May	Nov
Hoima							
*N*	369	59	92	37	27	142	94
HBR^a^	18.6	3.1	4.9	2.0	1.4	7.4	4.9
% contact indoors	98	99	99	99	99	99	99
EIR	0.74	0.05		0.15	0.15	0.19	0.21
Kyankwanzi							
*N*	3684	144	60	1471	406	2641	947
HBR^a^	197.1	7.5	2.9	78.9	21.7	141.6	50.2
% contact indoors	99	99	97	99	99	99	99
EIR	5.78	0.36		0.98	1.35	2.46	0.98
Kibaale							
*N*	25	71	26	68	50	54	76
HBR^a^	1.2	3.9	1.3	3.6	2.4	2.8	4.0
% contact indoors	95	100	98	99	96	98	98
EIR	0.00	0.10		0.00	0.00	0.09	0.00
Buliisa							
*N*	85	28	12	645	182	69	4
HBR*	4.0	1.4	0.6	33.8	8.4	3.4	0.2
% contact indoors	95	99	98	99	95	97	92
EIR	0.37	0.00		0.06	0.00	0.09	0.00
Buliisa	*An. funestus s.l.*						
*N*	189	22	1	11	19	0	204
HBR^a^	10.2	1.2	0.1	0.6	1.0		11.1
% contact indoors	100	99	100	100	100		100
EIR	0.21	0.00		0.00	0.00		0.00

^a^Calculated taking into account the assumed night time behaviour of the population

Sample sizes (N) for indoor and outdoor collections combined are indicated. Percent contact indoors was calculated by dividing the HBR indoors (18:00–06:00) over the total HBR indoors (18:00–06:00) and outdoors (18:00–22:00). In addition, the entomological inoculation rates (EIR; number of infective bites per person per night) are shown. The November 2010 collection EIR was not included due to a loss of samples

The sporozoite rates in *An. gambiae s.l.* varied between survey years and sites and were between 0.0-9.1 %, and a significant reduction over time was observed in Kyankwanzi and Buliisa (Table 4). Of the 112 *An. gambiae s.l.* samples positive for sporozoites, 111 were identified as *An. gambiae s.s.*, while one was *An. arabiensis*. The percentage of infected *An. funestus s.l.* in Buliisa was 2.0 % in 2009 while in subsequent years no infected individuals were observed, yet this change was not significant. When data were combined by species and study site, a significant reduction in sporozoite rates was observed during the duration of the study (*χ*^2^ = 17.42, *p* = 0.001), and sporozoite rates were 3.2 %, 3.0 %, 2.0 %, 1.8 % in 2009, 2010, 2011 and 2012, respectively.

**Table 4:** Percent of *An. gambiae s.l.* (all sites) and *An. funestus s.l.* (Buliisa site only) infected with *P. falciparum* sporozoites by survey year

Year	% sporozoite infected mosquitoes (number of samples tested)				
	*An. gambiae s.l.*				*An. funestus s.l.*
	Hoima	Kyankwanzi	Buliisa	Kibaale	Buliisa
2009	4.0 (325)	2.9 (3647)	9.2 (98)	0.0 (27)	2.0 (196)
2010	1.7 (60)	4.8 (145)	0.0 (35)	2.6 (77)	0.0 (25)
2011	9.1 (55)	2.9 (1221)	0.1 (793)	0.0 (125)	0.0 (29)
2012	3.3 (246)	1.8 (2885)	2.5 (81)	1.4 (145)	0.0 (195)
*χ* ^2^	4.99	12.42	62.35	3.47	5.13
Test	(*p* = 0.172)	(*p* = 0.006)	(*p* < 0.001)	(*p* = 0.324)	(*p* = 0.163)

The number of mosquitoes tested is indicated in brackets. Significant differences for each column were calculated using chi-square tests

**Fig. 3:**
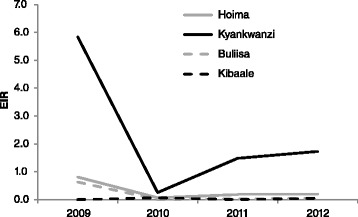
Entomological inoculation rates (EIR; number of infective bites per person per night) for *An. gambiae s.l.* and *An. funestus s.l.* combined over survey years, by site. The 2010 data point contains only data for May as the November round was not included due to a loss of samples

The entomological inoculation rate (EIR), i.e., the number of infective bites per person per night, was used to measure the intensity of malaria transmission in the four study sites (Fig. 3). A decrease in the EIR was observed in all sites compared with baseline figures for *An. gambiae s.l.*, with the exception of the Kibaale site. This reduction was most pronounced in the year following the UCC (Table 3). In Kibaale, the 2009 EIR was collected in a different site compared to following years, and the EIR in general was low in this site. In Buliisa, the EIR for *An. funestus s.l.* decreased after 2009.

### Feeding times and location

**Fig. 4:**
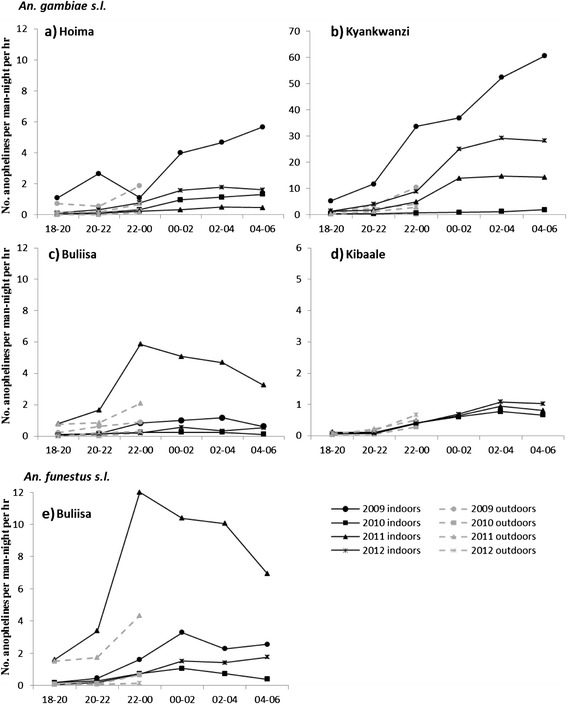
Human landing catch collection (indoor and outdoor) per 2 h intervals per site for 2009, 2010, 2011 and 2012. Data for *An. gambiae s.l.* (**a**-**d**) as well as *An. funestus s.l.* (**e**) for the Buliisa site is shown. The 2009 data were not presented for Kibaale as collections were done at a different site

The large majority of human-vector contact (> 80 % for almost all collections) took place indoors for all the years of the study (Table 3). Host-seeking behaviour patterns for *An. gambiae s.l.* showed that feeding took place throughout the night with the majority of bites occurring after midnight for most sites and years (Fig. 4). Feeding continued until the early morning hours of 04:00–06:00 h. *An. funestus s.l.* feeding patterns in Buliisa were similar and the majority of bites took place after midnight and activity remained high until the early morning hours, with the exception of 2011 where the peak feeding activity was between 22:00 and midnight.

The *An. gambiae s.l.* sample was dominated by *An. gambiae s.s.* in all sites except Buliisa where *An. arabiensis* was more prevalent and dominated outdoor collections (i.e., 59 %; Table 2). Indoors, this species was observed to be active throughout the night with a peak feeding time between 00:00–01:00 when 27 % of all bites were observed, while in outdoor collections the majority of feeding (29 %) occurred between 22:00–23:00 h.

Significantly more *An. gambiae s.l.* in Hoima (Z = 3.14; *p* = 0.002, *n* = 81) and Kyankwanzi (Z = 4.69; *p* < 0.001, *n* = 94) were caught indoors between the hours of six to midnight than outdoors over all collection rounds. In Kibaale, equal numbers were caught in both locations (Z = −1.01; *p* = 0.313, *n* = 49), while in Buliisa significantly more *An. funestus s.l.* were caught indoors during those hours (Z = 3.39; *p* = 0.001, *n* = 34). Similar numbers of *An. gambiae s.l.* in Buliisa were observed in indoor and outdoor HLC when considering the first half of the night (Z = 0.49; *p* = 0.627, *n* = 69).

### Insecticide resistance markers

A subset of *An. gambiae s.l.* specimens (N = 1086) were screened for the *kdr* L1014S mutation. In 2009, *kdr* L1014S frequencies were high in most sites in *An. gambiae s.s.* and varied between 70.8-85.4 % (Table 5). In Kibaale a lower frequency of 52.7 % was observed. In all sites, *kdr* L1014S frequencies increased significantly over the years and approached fixation in all sites in 2012 with the exception of Buliisa where the frequency was 86.0 % in 2012 down from 96.2 % in 2011. The *kdr* L1014S mutation was not observed in *An. arabiensis* with the exception of one heterozygous individual in Kyankwanzi in 2012. A much smaller number of samples were subsequently screened for the *kdr* L1014F mutation (47 for *An. gambiae s.s.* and 19 for *An. arabiensis* across study sites and years). Some *kdr* L1014F homozygous and heterozygous individuals were observed, and for all sites and years combined *kdr* L1014F genotype frequencies were 24.5 % for *An. gambiae s.s.* and 31.6 % for *An. arabiensis*.

**Table 5:** Percent *kdr* L1014S genotype frequency, number of mosquitoes tested (N), and percent homozygous resistant (RR) samples, per year and study site, for *An. gambiae s.s.* and *An. arabiensis*

Site	Year	*kdr* L1014S					
		*An. gambiae s.s.*			*An. arabiensis*		
		Frequency	*N*	RR	Frequency	N	RR
Hoima	2009	70.8	77	48.1			
	2010	78.8	26	69.2			
	2011	99.1	56	98.2	0.0	1	0.0
	2012	98.4	62	96.8			
	*p*-value	*χ* ^2^ = 69.68, *p* < 0.001					
Kyankwanzi	2009	85.4	103	73.8	0.0	1	0.0
	2010	87.9	29	75.9			
	2011	98.0	100	96.0	0.0	1	0.0
	2012	95.5	168	91.1	3.1	16	0.0
	*p*-value	*χ* ^2^ = 32.64, *p* < 0.001					
Buliisa	2009	72.4	38	57.9	0.0	16	0.0
	2010	86.7	15	73.3	0.0	8	0.0
	2011	96.2	52	94.2	0.0	58	0.0
	2012	86.0	43	79.1	0.0	37	0.0
	*p*-value	*χ* ^2^ = 19.18, *p* = 0.004					
Kibaale	2009	52.7	37	27.0			
	2010	60.3	29	31.0			
	2011	95.1	51	90.2			
	2012	99.2	62	98.4			
	*p*-value	*χ* ^2^ = 90.96, *p* < 0.001					

*P*-values compare genotype frequencies between years for *An. gambiae s.s*

## Discussion

The entomological surveys indicate that there was a reduction in malaria transmission intensity, measured by the EIR, in four high burden districts in Midwestern Uganda coinciding with a large scale increase in both coverage and use of LLINs and other antimalarial interventions. This decline was the result of both a decreased vector density and reduced infectivity of the vectors. The study was not designed to evaluate the effectiveness of the intervention in comparison with absence of such intervention. As no similar entomological data were collected in other areas, the potential contributions of other factors such as temporal variations in climate could not be assessed.

The decline in the EIR was most pronounced in the year following the LLIN UCC. As the area had high perennial malaria transmission, the campaign appears to have contributed to the reduction. Reductions in the EIR following deployment of vector control interventions were observed in a number of other settings [3, 29]. In subsequent years, an increase was observed in mosquito densities and the EIR, although values remained below the baseline figures. Ownership and use rates of nets in the project area declined due to a loss of nets as there was no additional campaign and only a limited number of LLINs were available through antenatal clinics. At the end of the project (approximately three years after the UCC), 64 % of households owned at least one ITN, and only 38 % of residents had slept under an ITN the previous night (Malaria Consortium, unpublished data). The decline in LLIN ownership due to attrition and deterioration of the nets in the years following the campaign may have contributed to the increased densities of vectors observed in the area. This highlights the importance of maintaining high levels of LLIN ownership and use following campaigns. Coverage with LLINs has recently been restored, as districts received nets as part of Uganda’s national universal coverage campaign in 2013.

The main vector species observed in all sites was *An. gambiae s.l.* Within this species complex, *An. gambiae s.s.* was identified as the dominant species in Kibaale, Hoima and Kyankwanzi, with *An. arabiensis* only observed at low frequencies in the latter two sites. In contrast, in Buliisa *An. arabiensis* was common, and in outdoor human landing catch collections this vector dominated, in line with the more exophagic nature of this species [30]. *An. funestus s.l.* was also primarily observed in Buliisa, with low numbers seen in Hoima and Kyankwanzi. *An. funestus s.s.* was the main species observed in the complex, but a small number of other species were also identified, i.e., *An. leesoni*, *An. parensis* and *An. rivulorum*. These species have all been previously identified from Uganda [31–34]. The Buliisa site was characterized by a complex species composition, and *An. arabiensis*, *An. gambiae s.s.*, and *An. funestus s.s.* were all observed. This site is situated on the shores of Lake Albert, and suitable larval habitats are found along the fringes of the lake, as well as inland for more temporary habitats. The relative species composition in this site varied across the study years, potentially due to the availability of suitable larval habitats and other factors. Vector densities were much higher in Kyankwanzi compared to the other study sites. Reasons for these high densities are not clear.

Vector densities declined in the light trap and human landing catch collections in the year following the UCC. Densities largely recovered in subsequent years, with seasonal peaks in the months of May and November following the rains, similarly to findings by Kilama et al. [35]. Although two different collection methods were used, the non-rotation of study houses between these methods did not allow for a direct comparison of trap efficiency. The daily EIR varied between 0–5.8 in 2009 to 0–0.98 in 2012 in this study. Prior to any scale up of interventions, the annual EIR in Uganda was determined in seven sites in 2001–2002 and varied between 4–1,568 infective bites per person with the highest EIR recorded in the Northern part of the country [36]. In a more recent study performed in 2011–2012, the estimated annual EIR varied between 4–125 depending on study site, and strong seasonal variation was observed [35]. Annual EIRs were not calculated here due to a lack of monthly data and the large seasonal fluctuations observed.

Extensive net use can result in behavioural changes of the vectors to earlier or outdoor feeding [6–9]. Before the scale up of LLINs in Uganda, *An. gambiae s.l.* was observed to mainly feed indoors after 11 pm in the majority of study locations [36]. Here, we did not observe any shifts in feeding behaviour to earlier times following the large scale introduction of LLINs. Throughout the study period, vectors were most active from midnight till 6 am when indoor collections ceased. The early morning feeding behaviour (i.e., 4–6 am) of both *An. gambiae s.l.* and *An. funestus* s.l. in most study sites was striking. In another recent study from the eastern region in Uganda, both *An. funestus* and *An. gambiae* were similarly active throughout the night until 6 am, although feeding rates remained more constant after midnight [37]. Further work is required to understand if feeding continues throughout the morning, as was recently demonstrated in *An. funestus s.s.* from Senegal [6]. Outdoor collections in this study were performed until midnight only. Although villagers are likely to be indoors after midnight and any outdoor biting preference after that time would have little relevance for malaria transmission, outdoor biting behaviour before dawn when inhabitants might be exposed due to early rising should be investigated further by including whole night biting collections outdoors.

The impact of LLINs on pyrethroid resistance was investigated by assessing *kdr* frequencies over time. The *kdr* L1014S mutation was observed at high frequencies in *An. gambiae s.s.* prior to the universal distribution of LLINs. The project areas had received ITNs as part of small targeted campaigns prior to 2010; additionally exposure to agricultural pesticides could have primed vectors [10]. *Kdr* L1014S frequencies increased in all sites and reached fixation in three sites, while in Buliisa frequencies increased to 96.2 % in 2011 but reduced to 86.0 % in 2012. Similar high *kdr* L1014S frequencies in *An. gambiae* s.l. were observed in another study from Uganda in 2011 [13]. Low *kdr* L1014S frequencies were observed in *An. arabiensis* (0–3.1 %), similarly to results from another study in Uganda [13]. The *kdr* L1014F mutation has not been observed at high frequencies in Uganda [13, 14, 12]. In this study, only a limited number of samples were screened for the *kdr* L1014F mutation. Some homozygous resistant *An. gambiae s.s.* and *An. arabiensis* were observed, but sample sizes were too small to deduce reliable estimates of frequencies per study site or assess trends over the years, and further studies are required.

No phenotypic resistance assays were undertaken as part of this study which would have allowed for a better understanding of the impact of *kdr* on resistance, but data collected in 2013 from the Kyankwanzi site showed phenotypic resistance to deltamethrin, permethrin, and DDT in *An. gambiae s.s.* in WHO tube assays (Malaria Consortium, unpublished data). Metabolic resistance mechanisms were not studied here, yet these could have also mediated pyrethroid resistance in these populations [14, 13].

### Limitations of the study

The aim of this study was to gather data on entomological variables to monitor changes in vector densities, behaviour and transmission intensity following the implementation of LLINs and other interventions. The study was not designed to investigate the effectiveness of these interventions in relation to a control situation where these interventions were not used. Therefore, the study is unable to assess the potential contributions of other factors that might affect transmission. Another limitation of the study was the lack of phenotypic resistance data in three of the four sites, as well as a more thorough assessment of other resistance mechanisms such as metabolic resistance.

## Conclusions

In conclusion, the entomological monitoring showed that malaria transmission intensity declined in the year following a large scale increase in coverage and use of LLINs and other antimalarial interventions in a high transmission area in Midwestern Uganda, but thereafter transmission started to increase again, although not to levels observed prior to the interventions. The increase could be due to decreased ownership and use of LLINs among others factors including vector resistance, temporal variations in climatic conditions, or a combination of these and other factors. The study showed that the distribution of LLINs did not result in changes in biting behaviour of the main vectors. Entomological surveillance of vector populations informs intervention performance and should be incorporated in routine monitoring of interventions.
